# SBE1 drives the circumferential growth of the starch sheath around the *Chlamydomonas reinhardtii* pyrenoid

**DOI:** 10.64898/2026.07.31.742166

**Published:** 2026-08-03

**Authors:** Micah I. Burton, Haoyu Wu, Lianyong Wang, Jessica H. Hennacy, Martin C. Jonikas

## Abstract

Pyrenoids are CO2-fixing organelles responsible for approximately one-third of global CO2 fixation. The pyrenoids of many algae are surrounded by a starch sheath proposed to perform the critical function of slowing leakage of concentrated CO2 out of the pyrenoid. How the cell shapes starch granules into curved starch plates that encase the pyrenoid to enable efficient CO2 fixation is currently unknown. Here, we elucidate how starch transitions from granules into a fully formed pyrenoid starch sheath using confocal microscopy of the model green alga *Chlamydomonas reinhardtii* . We observe that after initiation, starch granules grow circumferentially along the surface of the pyrenoid matrix to encapsulate it. We show that the starch branching enzyme SBE1 localizes to the pyrenoid and is essential for this circumferential starch granule growth. Our data suggest that SBE1 promotes the circumferential growth of pyrenoid-associated starch granules by branching starch at the matrix-granule-stroma interface. Our findings advance the understanding of pyrenoid starch sheath assembly and, more broadly, starch-shaping mechanisms.

**Significance Statement:**

The shape of the starch granules that surround the pyrenoid is critical for efficient carbon fixation, but how these granules are shaped into curved plates is currently not understood. We find that starch granules spread along the pyrenoid surface rather than growing equally in all directions, and that this bias requires SBE1, a branching enzyme localized to the surface of the spherical pyrenoid matrix condensate. The findings support a model in which localizing a metabolic enzyme directs polymer growth to create curvature. This mechanism connects branching enzyme placement to starch granule geometry and contributes to the understanding of how cells convert local biosynthetic activity into large-scale organelle architecture.

## Full Text Availability

The license terms selected by the author(s) for this preprint version do not permit archiving in PMC. The full text is available from the preprint server.

